# The effect of Wenyang Huoxue decoction combined with Baduanjin exercise on the cardiac function and quality of life of patients with Yang deficiency coronary heart disease complicated with chronic heart failure

**DOI:** 10.1097/MD.0000000000039876

**Published:** 2024-12-27

**Authors:** Miao Zhou, Zhi-Kun Lai, Feng Tao, Hao-Xuan Ni, Xiao-Zhen Hu

**Affiliations:** a Department of Internal Medicine-Cardiovascular, Shanghai Municipal Hospital of Traditional Chinese Medicine, Shanghai University of Traditional Chinese Medicine, Shanghai, China; b Department of Endocrinology, Shanghai Municipal Hospital of Traditional Chinese Medicine, Shanghai University of Traditional Chinese Medicine, Shanghai, China.

**Keywords:** Baduanjin, chronic heart failure, coronary heart disease, Wenyang Huoxue decoction

## Abstract

**Background:**

Heart failure (HF) caused by coronary heart disease (CHD) is becoming increasingly common, and it still has a high morbidity and mortality rate. Previous studies have found that Wenyang Huoxue decoction combined with Baduanjin exercise can improve the cardiopulmonary function of patients with CHD, but it is not clear whether the heart function of patients with CHD complicated with HF can be improved by this treatment. This study aimed to evaluate the clinical effect of Wenyang Huoxue decoction combined with Baduanjin exercise on CHD complicated with HF.

**Methods:**

Patients with HF who received outpatient and inpatient treatment in the Shanghai Hospital of Traditional Chinese Medicine between 2019 and 2021 were enrolled in this study. The patients were randomly divided into a control group and a treatment group. N-terminal prohormone of brain natriuretic peptide, soluble growth stimulating express gene 2, stroke volume index, Minnesota Living with Heart Failure Questionnaire scores, and cardiac ultrasound were observed after 12 weeks of treatment.

**Results:**

100 patients were included, 3 were lost to follow-up, 49 in the treatment group and 48 in the control group. With a 30% reduction of N-terminal prohormone of brain natriuretic peptide as the primary endpoint, the effective rate of the treatment group was 57%, while that of the control group was 35%. In the meanwhile, stroke volume index and ejection fractions increased significantly, while soluble growth stimulating express gene 2 and Minnesota Living with Heart Failure Questionnaire scores were significantly decreased in the treatment group after treatment. There was no significant improvement trend in the control group.

**Conclusions:**

This study confirmed that treatment with Wenyang Huoxue decoction and Baduanjin exercise can improve the cardiac function and quality of life of patients with CHD complicated with HF and that it may have certain advantages in improving ventricular remodeling.

## 1. Introduction

Heart failure (HF) is a clinical syndrome in which the pumping and/or filling function of a ventricle is reduced due to a variety of causes. It is characterized by difficulty breathing and/or limited movement and has high morbidity and mortality rates. According to an epidemiological survey conducted in China in 2003, the prevalence rate of HF in adults aged 35 to 74 was 0.9%.^[1]^ Data from Europe showed that the 1-year mortality rate of HF was 7.2%,^[2]^ the 5-year mortality rate was 75.3%, and the readmission rate was 82.2%.^[3]^ With the increasing aging of the population, HF has become a serious health issue. Coronary heart disease (CHD) accounts for 49.6% of the complications of HF and is one of the main complications of the syndrome.^[4]^

Traditional Chinese medicine (TCM) believes that Yang deficiency and blood stasis are the main pathological changes of HF. Wenyang Huoxue decoction combined with Baduanjin exercise has been proven to improve the cardiopulmonary function and quality of life of patients with CHD, as well as their peak oxygen uptake.^[5]^ Peak oxygen uptake is an indicator of cardiac insufficiency, and low peak oxygen uptake is one of the indicators of poor prognosis in patients with HF.^[6]^ Additionally, multiple clinical evidences have demonstrated that Baduanjin exercise could significantly improve heart function, for example, preventing post-myocardial infarction left ventricular remodeling,^[7]^ enhance of cardiorespiratory endurance^[8]^ as well as showing beneficial effects on chronic HF.^[9]^ However, there have been no evidence-based studies showing that Wenyang Huoxue decoction combined with Baduanjin exercise can treat HF. The present study aimed to expand the research into the use of Wenyang Huoxue decoction combined with Baduanjin exercise to treat CHD and conduct a randomized controlled study to evaluate the clinical effect of Wenyang Huoxue decoction in the treatment of HF caused by CHD.

## 2. Methods

### 2.1. Study design

This study adopted a randomized, placebo-controlled method. It could not be double blind due to the nature of kung fu exercise, so in order to reduce the bias caused by single blindness, research center control and group management were adopted: the Baduanjin exercise was led by a therapist, medication distribution was performed by a clinical pharmacist, and data collection and reduction was undertaken by a physician. The general manager was responsible for random grouping; they did not directly participate in the treatment and follow-up of patients but summarized the data at the end of the study. The general manager had the right to access all the information about a patient, while other managers were not allowed to inquire about a patient’s treatment plan.

This study was conducted with approval from the Ethics Committee of the Shanghai Hospital of Traditional Chinese Medicine (batch number: 2021SHL-KY-73). Written informed consent was obtained from all participants.

### 2.2. Sample size calculation

Based on previous experimental results,^[5]^ the difference between the experimental group and the control group was 2.24 with a standard deviation of 2.56. With a two-sided α = 0.05 and a power (1 − β) of 0.9, and assuming an equal sample size ratio of 1:1 between the experimental and control groups, we used R language to calculate the required sample size according to the method by Chow et al.^[10]^ This calculation indicated that 28 subjects were needed in each group.

Considering a potential 20% dropout rate, at least 35 subjects were needed in the experimental group and 35 in the control group, totaling a minimum sample size of 70. Given the diversity of the intervention factors (2 intervention factors), to ensure patient compliance and data completeness, we planned to recruit 100 patients in total.

The formula for calculating sample size is $n=\frac{{\left(Z_{\alpha }+Z_{\beta }\right)}^{2}\times 2\sigma^{2}}{\delta^{2}}$.

### 2.3. Inclusion and exclusion criteria

All patients were outpatients or inpatients of the Shanghai Hospital of Traditional Chinese Medicine.

Inclusion criteria: patient aged 18 to 75; CHD confirmed by previous coronary angiography, coronary computed tomography angiography, or treadmill exercise test, or the patient had a clear history of myocardial infarction and angina pectoris; patient’s HF was New York Heart Association grade Ⅲ; N-terminal prohormone of brain natriuretic peptide (NT-proBNP) of the patient’s sinus heart rate within 4 weeks was greater than 1000 pg/mL, and NT-proBNP of the patient’s atrial fibrillation within 4 weeks was greater than 1600 pg/mL; and patient provided informed consent.

Exclusion criteria: patient younger than 18 years of age or older than 75 years of age; acute coronary syndrome; patient had serious liver and kidney function injury, hematological system diseases, or serious infectious diseases; acute infectious diseases, including tuberculosis, AIDS, and acute-stage hepatitis; patient was pregnant or lactating; patient was taking other TCM herbal compounds or medicine decoctions for treatment; the patient performed Baduanjin, Tai Chi, or other traditional kung fu exercises in the past month; and the patient had severe pulmonary insufficiency and physical disability and could not undergo a cardiopulmonary exercise test.

### 2.4. Diagnostic criteria

The diagnosis of HF was made according to the Chinese Guidelines for the Diagnosis and Treatment of Heart Failure 2018.^[11]^ Patients at stage C, which is clinical HF – had organic heart disease, past or present symptoms, and were at grades Ⅰ to Ⅲ according to the New York Heart Association scale.

Patients were diagnosed with Yang deficiency if they had a combination of the following symptoms with a total score ≥ 8: a chilly sensation with cold limbs (3 points); dyspnea after activity (3 points); loose stools (2 points); frequent nocturia (2 points); abdominal and lumbar cold (2 points); a light-colored, fully filled, and moist tongue (2 points); a deep pulse (2 points).

### 2.5. Treatment, intervention, and grouping

All patients were given basic care according to HF guidelines. The control group received TCM placebo treatment, while the Wenyang Huoxue decoction and Baduanjin exercise group (treatment group) undertook Baduanjin exercise and received Wenyang Huoxue decoction.

#### 2.5.1. Wenyang Huoxue decoction granula

The Wenyang Huoxue decoction granule consisted of aconite, chiretta, cattail pollen, root of red or white peony, dried fruit of citron or trifoliate orange, and root of balloon flower. The decoction was prepared into a formula by Jiangsu Tianyin Pharmaceutical using the same amount of crude drug, which was processed by boiling and dehydration. The dose was 72 g twice a day. The placebo given to the control group consisted of 10% crude drug plus excipient.

#### 2.5.2. Baduanjin exercise^[12]^

The exercise is divided into 8 movements: propping up the sky; drawing the bow; raising one hand; looking over the shoulders; clenching fists and looking forward with eyes wide open; pulling toes; swaying head and buttocks; and jolting. The target rate was determined by the anaerobic threshold (AT), with 80% heart rate at AT being the target and exercise intensity not exceeding the target rate.

Patients were instructed to undertake Baduanjin exercise for 10 minutes 3 times a week initially, gradually increasing the frequency and duration of workouts to a maximum of 30 to 60 minutes 5 times a week. The workouts then lasted no less than 30 minutes each time for 12 weeks, with each workout consisting of 5 minutes of warm-up, 25 minutes of exercises, and 5 minutes of relaxation.

### 2.6. Measurement indexes

#### 2.6.1. Peak oxygen uptake (VO_2_ peak)

Peak oxygen uptake data was obtained through symptom-limited cardiopulmonary exercise testing (CPET). After resting for 3 minutes, a no-load warm-up was performed at a pedaling rate of (60 ± 5) r/min for 3 minutes, after which the patient began a load period of increasing the ramp power (20 w/min). The Borg scale was used to identify and record the fatigue level of patients reaching symptom-limited CPET. The exercise test indexes under peak were obtained using a telemetry cardiopulmonary tester, and recovery data beyond 6 minutes continued to be recorded. Finally, the patient’s heart rate, blood pressure, electrocardiogram, and blood oxygen saturation were monitored until a normal physiological state was restored. K4B2 version 10.0 software analysis and V-slope^[11]^ were used to evaluate CPET motion test indexes under AT.

#### 2.6.2. NT-proBNP

A Lenew800 colloidal gold test strips analyzer was used for rapid detection, and fingertip blood was taken for analysis.

#### 2.6.3. Stroke volume index (SVI)

The BioZ.com^TM^ digital noninvasive hemodynamic monitoring system was adopted, and a cardiac resistance meter was used for measurement. Measurements were taken before and after treatment. Stroke volume (SV) referred to the amount of blood expelled from one ventricle during a heartbeat. SVI was the result of SV divided by body surface area based on height and weight.

#### 2.6.4. Soluble growth stimulating express gene 2 (sST2)

To use it as a measurement index, a total of 5 ml of fasting blood was collected from patients, and an ST-360 automatic microplate reader was used for analysis.

#### 2.6.5. Minnesota Living with Heart Failure Questionnaire (MLHFQ)

A Chinese translation of the MLHFQ was adopted. Patients scored themselves before and after 12 weeks of treatment, and the scores were calculated based on total scores.

#### 2.6.6. Cardiac ultrasound

Conventional cardiac color Doppler, ask the same ultrasound doctor to measure the left ventricular ejection fraction and left ventricular end diastolic diameter of all patients before and after treatment.

### 2.7. Drug combination

The use of hypertensive drugs, diabetes drugs, anticoagulants, antiplatelet drugs, or statins before enrollment remained unchanged. Patients taking cardiac glycosides and diuretics continued to take them. Patients not taking diuretics were advised not to start taking them during treatment. Any drug changes during treatment were recorded.

### 2.8. Statistical analysis

SPSS 25.0 software was used for statistical analysis. Gender, cardiac function grading, and number of complications (expressed as a percentage) were evaluated using a χ^2^ test. Normally distributed data including height, weight, and scores of MLHFQ were expressed as mean ± standard deviation. Non-normally distributed data, including NT-proBNP, were expressed as a median (upper quartile–lower quartile). Normally distributed data were statistically analyzed using 2 independent sample *t* tests or a non-parametric test. Additionally, analysis of covariance was performed to investigate the impact of Wenyang Huoxue decoction combined with Baduanjin exercise on the indicators including PVO_2_, SVI, and ST2 of patients with Yang deficiency coronary heart disease complicated with chronic heart failure, and the data were expressed as marginal means ± SE for Normally distributed data. *P* < .05 was considered statistically significant.

## 3. Results

### 3.1. Baseline data

It was initially planned that each group would consist of 50 patients. This was the case after initial enrollment. However, one person in the treatment group was lost to follow-up due to the inability to follow up continuously. In the control group, one patient was hospitalized during the treatment period and took additional cardiac agents, thereby being excluded, while one patient was lost to follow-up. A total of 49 patients in the treatment group (23 males and 26 females) and 48 patients (19 males and 29 females) in the control group were finally included in the statistical analysis.

Because of the need for exercise testing and exercise interventions, the population included was relatively young. There were no significant differences in age, height, or weight between the 2 groups.

Patients with stable CHD at grade II to III cardiac function were selected. The treatment group included 23 patients with grade II cardiac function and 26 patients with grade III cardiac function, while the control group consisted of 18 patients with grade II cardiac function and 30 patients with grade III cardiac function.

In the treatment group, 9 patients were complicated with atrial fibrillation, 24 with hypertension, and 13 with diabetes. In the control group, 29 patients were complicated with hypertension, 12 with atrial fibrillation, and 10 with diabetes (some patients had more than one complication). There was no significant difference between the 2 groups in comorbidity. Besides, NT-proBNP was 1807 (1079–2955) pg/mol in the treatment group and 1460 (952–2367) pg/mol in the control group before treatment. There were no significant differences in any of the indicators between the 2 groups (see Table 1).

**Table 1:** Baseline levels in 2 groups

n	Treatment group	Control group	*P*
	49	48	
Man	23	19	.541
Female	26	29	
Age	67.78 ± 7.78	66.56 ± 10.79	.526
Height	164.51 ± 7.414	164.42 ± 8.66	.955
Weight	67.57 ± 12.05	64.96 ± 11.819	.284
Cardiac function grade I	13	8	.491
Cardiac function grade II	27	25	
Cardiac function grade III	13	11	
Combination with atrial fibrillation	9	12	.428
Combination with hypertension	24	29	.258
Combination with diabetes	13	10	.680
NT-proBNP (pg/mol)	1807 (1079–2955)	1460 (952–2367)	.146

### 3.2. Treatment effect

The treatment group received 12 weeks of treatment with Wenyang Huoxue decoction and standard sufficient Baduanjin exercise. The control group maintained the original treatment regimen, receiving a TCM placebo but making no lifestyle changes. The original combined medication of the 2 groups is shown in Table 2, and there is no statistical difference.

**Table 2:** Combined medication

	Treatment group (n = 49)	Control group (n = 48)	*P*
Beta blocker	40	38	.36
ACEI/ARB	38	34	.449
ARNI	10	10	.959
Diuretic	38	40	.473
Aldosterone blocker	16	20	.358
SGLT2	14	11	.524

An NT-proBNP decrease of 30% was regarded as indicating effective treatment. The effective rate was 35% in the control group and 57% in the treatment group, the difference between which was statistically significant (*P* < .05; see Table 3). This suggests that Wenyang Huoxue decoction is effective in treating HF.

**Table 3:** A 30% reduction in NT-proBNP as effective rate to calculate the treatment effective rate

	Ineffective	Effective	Total	Effective rate
Treatment group	21	28	49	57%
Control group	31	17	48	35%
Total	32	65	97	*P *= .032

Cardiopulmonary function was assessed before and after treatment. VO2 peak in the treatment group increased from (15.24 ± 2.98) to (17.42 ± 3.34) mL/kg/min, which was a statistically significant increase, while in the control group it increased from (16.06 ± 3.67) to (16.48 ± 3.76) mL/kg/min, which was not statistically significant. The peak oxygen uptake of the treatment group was higher than that of the control group (see Table 4).

**Table 4:** Changes of peak oxygen uptake, SVI and sST2 indicators before and after treatment in both control and treatment groups

Indicators	Before or after treatment	Control group	Treatment group	*P1*	*P2*	*P3*	*P4*
VO_2_ peak (mL/kg/min)	Before treatment	16.06 ± 3.67	15.24 ± 2.98	0.075	0.166	0.875	0.003**
	After treatment	16.48 ± 3.76	17.42 ± 3.34				
SVI (mL/beat/m2)	Before treatment	35.52 ± 2.70	35.21 ± 4.00	0.629	0.000**	0.610	0.000**
	After treatment	35.85 ± 2.56	38.94 ± 2.91				
sST2	Before treatment	34.38 ± 5.36	32.24 ± 3.96	0.165	0.001**	0.237	0.001**
	After treatment	31.57 ± 7.98	27.19 ± 7.41				

*P*1 value represented the comparison between control and treatment group before treatment, *P*2 value represented the comparison between control and treatment group after treatment, *P*3 represented the comparison before and after treatment in control group, *P*4 value represented the comparison before and after treatment in treatment group.

** *P*2 < 0.001 vs control group after treatment and

**P4 < .001 vs treatment group before treatment.

SVI of treatment group was (35.21 ± 4.00) mL/beat/m^2^ before treatment and (38.94 ± 2.91) mL/beat/m^2^ after treatment. The difference before and after treatment in the treatment group as well as the difference between control and treatment group were both statistically significant (*P* < .001), but the difference in the control group was not (see Table 4).

sST2 was (32.34 ± 3.96) ng/mL in the treatment group and (34.38 ± 5.36) ng/mL in the control group before treatment. There were no significant differences between the 2 groups in baseline sSt2 values. After treatment, the sST2 value of the control group became (31.57 ± 7.98) ng/mL with no significant differences. While sST2 value of the treatment group decreased to (27.19 ± 7.41) ng/mL. Although the decrease was small, the sST2 value performed statistic difference in treatment group after treatment as compared as that of value before treatment (*P* < .001). Of note, a statistic difference of sST2 value could been showed between control and treatment groups after treatment (*P* < .001, see Table 4).

A total of 100 MLHFQ forms were distributed before treatment, and 100 were collected. After treatment, 97 forms were distributed, and 97 were collected. The MLHFQ score was (38.98 ± 9.85) in the treatment group and (40.88 ± 9.73) in the control group before treatment, with no significant difference between the groups. After treatment, the quality of life of the treatment group was significantly improved and the MLHFQ score decreased to (32.78 ± 8.21), which was statistically significant. The control group did not improve significantly. The life satisfaction of patients in the treatment group also increased significantly (see as Table 5).

**Table 5:** Minnesota living with heart failure questionnaire

	Before treatment	After treatment	*P* value of paired *t* test before and after treatment
Treatment group	38.98 ± 9.85	32.78 ± 8.21	0
Control group	40.88 ± 9.73	40.54 ± 9.51	.383
*P* value between groups	0.345	0	

There was no significant difference in ejection fractions and left ventricular end diastolic diameter between the 2 groups before treatment, but the ejection fractions of the treatment group was improved after treatment. The left ventricular end diastolic diameter has not been significantly improved (see Table 6).

**Table 6:** Cardiac ultrasound data

		n	Mean	*P* value
EF Before treatment	Treatment group	49	45.24 ± 10.29	.386
	Control group	48	43.50 ± 9.43	
EF After treatment	Treatment group	49	49.06 ± 9.22	.001
	Control group	48	43.06 ± 8.81	
LVED Before treatment	Treatment group	49	55.10 ± 7.38	.263
	Control group	48	57.02 ± 9.31	
LVED After treatment	Treatment group	49	55.35 ± 7.22	.213
	Control group	48	57.42 ± 8.97	

EF = ejection fractions,

## 4. Discussion

As the treatment strategy of acute coronary syndrome becomes more efficient, the mortality rate of this disease continues to decrease, resulting in an increase in the number of patients with cardiac insufficiency caused by CHD. However, the death rate of HF remains a high level. Cardiac rehabilitation methods exhibited an effect on improving quality of life and reducing mortality and disability rates by following up the changes of a patient’s condition and intervening in their life in multiple ways, including psychology (e.g. education^[13]^), exercise, water intake, smoking cessation, medication and taking appropriate intervening for patients with certain characteristics.^[14]^

Wenyang Huoxue decoction is a TCM compound formulation that is extracted from several plant medicines through common decoction processing. It that has been used as a decoction in the Shanghai Hospital of Traditional Chinese Medicine for many years. It was originally used for the treatment of patients within a year after cardiac intervention, but it has been gradually extended to the treatment of various types of CHD in recent years. However, according to TCM theory, only patients with traditional Yang deficiency and blood stasis can use this medicine. According to the Chinese guidelines for stable CHD,^[15]^ this study selected a chilly sensation with cold limbs (3 points), dyspnea after activity (3 points), loose stools (2 points), frequent nocturia (2 points), abdominal and lumbar cold (2 points), a light-colored, fully-filled, and moist tongue (2 points), and a deep pulse (2 points) as symptoms of Yang deficiency; on the basis of the etiology of CHD and HF, a combination of these symptoms scoring ≥ 8 points was taken as the basis for differentiation and inclusion. In TCM, blood stasis refers to a condition in which the blood does not flow smoothly. According to this theory, blood stasis syndrome runs through the whole process of HF in CHD, so only Yang deficiency syndrome was used in the inclusion criteria.

Additionally, exercise rehabilitation is the main palliative method of cardiac rehabilitation, but the currently recommended cardiac exercise rehabilitation process is complicated and difficult to implement, which is not conducive to promotion. As a light to moderate physical activity, Baduanjin is a traditional form of Chinese kung fu that is light to moderate in intensity^[16]^ and can be undertaken by patients with any level of athletic ability. Therefore, patients with grade Ⅱ–Ⅲ cardiac function were selected for exercise. Baduanjin is a nationally known method of exercise combining physical activity, breathing, and psychological adjustment. It is gentle, slow, flexible, and coherent, simultaneously loose and tight, according to the theory of association of activity and inertia of traditional Chinese medicine, and combining aerobic exercise and breathing exercise, which can increase the chest volume, reduce the compression of the heart and lungs, and promote the blood circulation in the chest and abdominal cavity.^[17]^ Patients with Yang deficiency easily form phlegm and blood stasis, resulting in blood blockage. Baduanjin can play a role in clearing and activating the channels and collaterals, raising Yang and strengthening the spleen through movement-guided exercise. At the same time, Baduanjin’s slow rhythm can relieve tension and anxiety, soothe the liver, and regulate Qi, which is similar to the effects of the Wenyang Huoxue decoction. However, Yang-Qi is the main substance that promotes blood movement. Patients with Yang deficiency show symptoms of wheezing and chills similar to those of cardiac insufficiency, and these symptoms can be alleviated by warming the Yang and vitalizing the blood.

Wenyang Huoxue decoction follows the academic theory of treating the Qi and blood together, combining the warming of the Yang and vitalizing of the blood with the balancing of the Qi and blood, Yin and Yang. Aconite in the decoction can enhance Yang, supplement Qi, and promote blood circulation, it also has a cardiotonic effect, and its decoction can improve the hemodynamics of patients with HF after CHD.^[18]^ As assistant medicines, chiretta, cattail pollen, and the root of red or white peony can nourish blood and promote blood circulation. Dried fruit of citron or trifoliate orange and root of balloon flower play an important role in the recovery of overall Qi and can increase or reduce the movement of vital Qi. Chiretta and cattail pollen belong to the homology of medicine and food and are also used as soup ingredients in China. The combination of dried fruit of citron or trifoliate orange and the root of balloon flower is a combination used in the classic Xuefu Zhuyu decoction. This prescription has achieved good results in rehabilitation after percutaneous coronary intervention and has been found to significantly improve the symptoms and quality of life of patients.^[19]^

Preliminary tests have confirmed that Wenyang Huoxue decoction combined with Baduanjin exercise can improve cardiopulmonary function in patients with CHD. Patients with CHD complicated with HF were included in this study for further research. Due to the nature of exercise, however, it was not possible to adopt a double-blind method. The compliance of patients in the treatment group was relatively good.

Noninvasive hemodynamic monitoring is a simple method that can quickly measure the cardiac function of patients, and SVI can reflect this cardiac function. In the present study, SVI increased significantly in the treatment group. There are 2 possible reasons for this increase: enhancement of myocardial contractility and increase of returned blood volume.^[20]^ The selected patients had stable HF and stable body fluid levels, so the improvement of SVI could also be attributed to the improvement of ventricular systolic and diastolic functions.

Peak oxygen uptake is an important prognostic indicator of HF. Peak oxygen uptake below 1 mL/kg/min is an indication of heart transplantation and poor prognosis.^[21]^ Cardiac function can be classified into 4 levels based on peak oxygen uptake: grade A > 20 mL/min/kg, no or mild cardiac insufficiency; grade B = 16–20 mL/min/kg, mild to moderate cardiac insufficiency; grade C = 10–15 mL/min/kg, moderate to severe cardiac insufficiency; grade D < 10–15 mL/min/kg, severe cardiac insufficiency.^[17]^ Most of the patients included in this study were grade B patients with fair mobility, so they were able to persist in exercise. After 12 weeks of observation, Wenyang Huoxue decoction and exercise was found to significantly improve the cardiopulmonary function of patients.

The MLHFQ is an important measure of quality of life in patients with HF. The scale includes a somatic state score and an emotional state score. After a period of treatment, the quality of life of patients with HF improved significantly, which was consistent with an increase in peak oxygen uptake and an increase in activity tolerance. At the same time, gentle long-term exercise also reduced patients’ negative emotional states. This beneficial effects on above aspects of Baduanjin have been also reported by previous literatures.^[22]^

sST2 is a member of the interleukin-1 receptor family. When the myocardium is stimulated by stretch, ST2 (which includes sST2 and transmembrane ST2[ST2L], another subtype of ST2) is significantly up-regulated, and sST2 is more obviously up-regulated, so it easily competes with ST2L, binds to interleukin-33, and blocks cardiac protection.^[19]^ sST2 is associated with poor prognosis in elderly patients with systolic and ischemic HF.^[23]^ The patients enrolled in the present study had relatively stable conditions, and the overall sST2 level was not high. However, the study observed a downward trend in sST2 in the treatment group. Therefore, the effect of Wenyang Huoxue decoction combined with Baduanjin on the body’s inflammation system needs further study.

The present study had some limitations. First, it did not follow the requirements of double blindness. However, a series of measures were taken to compensate. Second, according to TCM theory, Wenyang Huoxue decoction cannot be given to all patients with CHD complicated with HF, and patients should be screened strictly according to the criteria of syndrome differentiation. Third, the number of cases included in this study was relatively small, and the correlation between Baduanjin’s different exercise times and various biochemical index levels was not studied. Future research with a larger sample is required.

## 5. Conclusion

This study demonstrated that treating with Wenyang Huoxue decoction and Baduanjin exercise can improve the cardiac function and quality of life of patients with CHD complicated with HF and that it may have certain advantages in improving ventricular remodeling, suggesting that a combination of TCM and Baduanjin exercise could be used as an important treating strategy of cardiac rehabilitation, as it is safe and nontoxic and can efficiently treat this disease.

## Acknowledgments

We would like to acknowledge the hard and dedicated work of all the staff that implemented the intervention and evaluation components of the study.

## Author contributions

**Conceptualization:** Miao Zhou, Feng Tao, Xiaozhen Hu.

**Data curation:** Miao Zhou, Zhi-Kun Lai, Xiaozhen Hu.

**Formal analysis:** Miao Zhou, Zhi-Kun Lai, Hao-Xuan Ni.

**Funding acquisition:** Xiaozhen Hu.

**Methodology:** Miao Zhou, Zhi-Kun Lai, Hao-Xuan Ni.

**Writing – original draft:** Miao Zhou, Zhi-Kun Lai, Hao-Xuan Ni.

**Writing – review & editing:** Xiaozhen Hu.
